# Dry Fibre Placement: The Influence of Process Parameters on Mechanical Laminate Properties and Infusion Behaviour

**DOI:** 10.3390/polym13213853

**Published:** 2021-11-08

**Authors:** Benjamin Grisin, Stefan Carosella, Peter Middendorf

**Affiliations:** Institute of Aircraft Design (IFB), University of Stuttgart, Pfaffenwaldring 31, 70569 Stuttgart, Germany

**Keywords:** dry fibre placement, mechanical properties, carbon fibre, gaps, VAP, thermoset, binder, fixed tow

## Abstract

Within the dry fibre placement (DFP) process, spread and pre-bindered carbon fibre rovings are automatically processed into dry textile preforms using 2-D and 3-D laying systems. The aim was to automate existing hand lay-up processes, reducing the complexity, increasing robustness, and facilitating the handling of the DFP technology. Process reliability, low waste rates, and flexible production are demonstrated. In this publication, the influences of the process parameters, 2 mm wide gaps and the percentage of 90° plies in the laminate, are investigated with regard to the mechanical properties, the permeability, and the infusion times in the preform z-direction (thickness). The effects on stiffness and strength are compared for several use cases. An approach to determine the infusion times as a function of the laminate thickness, the ply structure, and 2 mm wide gaps is demonstrated and analysed using vacuum-assisted process (VAP) infusion tests. The investigations are performed with carbon fibre tows (24 k), a reactive epoxy-based binder system, and a thermoset infusion resin system.

## 1. Introduction

Fibre-reinforced plastic composites are created by combining reinforcing fibres with polymers, which serve as matrix. If the fibres are first combined with the matrix and then moulded into shape, this is called a prepreg process. If the dry fibres are first shaped into a so-called preform and then impregnated with a matrix system, it is called the preforming process. One of the preforming processes is a dry fibre placement (DFP) process called crosslayer. The production of fibre composite structures from spread dry carbon fibre tows, so-called fixed tows [1], is pursued with the aim of producing homogeneous laminates with a minimum of defects. The main advantages of DFP are low fibre undulation and low fibre areal weights (FAWs) within the plies. However, the low permeability of the preforms produced is a major disadvantage [2]. It is possible to specifically control the required FAW of the individual plies of a laminate. The FAW of a single ply is adjusted by the spreading width of the tows and the web spacing. According to Gardiner [3], 80% of the carbon fibres are currently spread before processing. Bindered and spread fibres can be placed in any orientation within a single ply. Alternatively, the tows can be placed within a defined area to realize local reinforcements. Therefore, the preform structure can be designed to be load-path oriented and with a near-net-shape. Depending on the requirements and layup technology, the fibre deposition can be made on plane and on three-dimensional moulds. The scope of this research is an evaluation the DFP process chain in combination with a simple state of the art liquid resin infusion method (VAP).

### 1.1. Fibre Placement State of the Art

In the field of fibre placement techniques, there are two main categories: the automated fibre placement (AFP) and the automated tape laying (ATL) technique. AFP is a mostly robot-based placement method of spread, non-impregnated, but bindered rovings. The used fixed tows can have widths from about 3 to 25 mm. The most commonly applied widths are 3.2 (1/8″), 6.4 (1/4″), and 12.7 mm (1/2″) [4]. The laying heads are equipped with up to 32 individually controlled fibre feeders, which can be controlled and cut individually. The process is suitable for very complex and multi-curved components and can take place on concave and convex tool shapes, which can also represent complex geometries. Due to the individually controlled fibre paths, the outer contour of a component can be mapped very precisely, which leads to little waste. Furthermore, the formation of wrinkles on multi-curved surfaces is significantly reduced. Therefore, the DFP process used in this work can be classified as AFP. ATL is a process in which wide prepreg tapes (up to 300 mm) are automatically laid usually using very large gantry systems. Slightly concave or convex shapes can be covered. The complexity of the part geometry can be classified as low to medium. Typical applications, for example, are outer skin shells of aircraft components, like fuselage segments or wing shells. The advantages are a large deposition rate and very large component geometries. Disadvantages include the high investment costs and reduced deposition rates with increasing component complexity [4]. Small- and medium-sized companies often have applications besides aerospace, with lower structural and material requirements for their applications. In this case, it is often not economically viable to use this type of machine [5].

**Figure 1:**
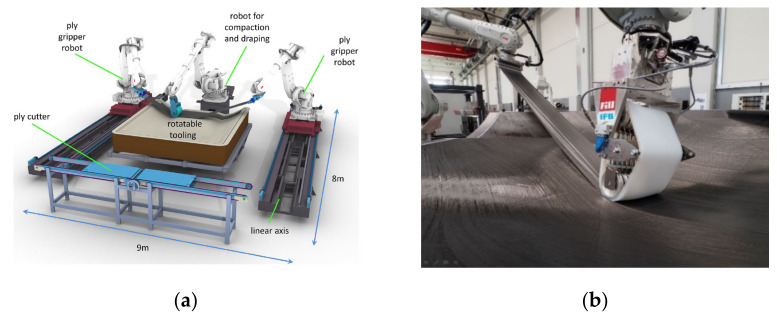
(**a**) APP principle [7]; and (**b**) APP end effector [7].

Since ATL/AFP are already well established, the recent developments in the field of fibre placement focus on alternative or simplified approaches for the machine technology like the APP (advanced ply placement). Within the APP process, three coordinated robots imitate the manual placement of wide unidirectional fabrics. Two gripper robots hold the material to be deposited and a third robot, equipped with a shape adaptive application roller, applies it to the mould, see Figure 1a,b. Prepreg materials, bindered tapes, and bindered semi-finished products can be used too [5]. Besides the development of new machine types, another focus is the improvement of the existing machines. Fibre steering is a recent topic of interest. The aim is the realisation of curved fibre paths by shearing the spread tow. In [6], it is described that dry tows show better shearing behaviour than pre-impregnated tows. Realising curved paths can also increase the thickness homogeneity and decrease process-induced defects during layup, e.g., fibre buckling or tow overlaps [6].

### 1.2. Impact of Preform Defects on Mechanical Properties

The quality of a DFP preform is described considering the following evaluation criteria:

- Gaps.
- Overlaps.
- Fibre undulation/waviness.
- Wrinkles.
- Fuzz balls/fibre fluff.
- Binder residue.
- Loose fibre (surface/tow edges).
- Fibre folding.
- Twisted tows.

**Figure 2:**
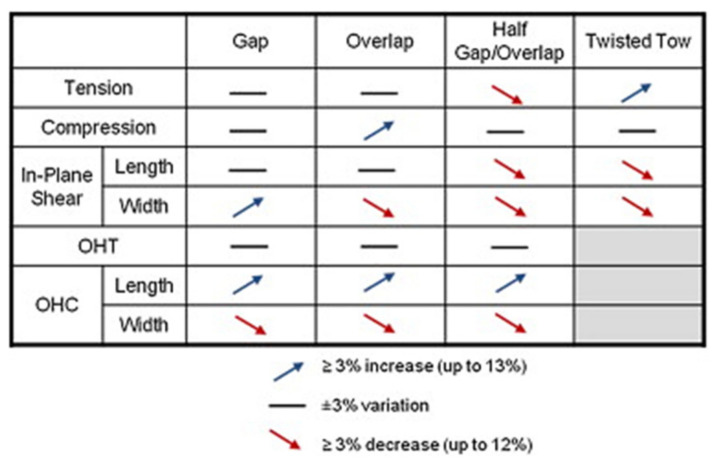
Overview of layup defects on the strength of AFP prepreg laminates, according to [8], OHT (open hole tension), OHC (open hole compression).

Croft et al. [8] describe that the influence of gaps and overlaps on the mechanical tensile and compressive strength of a prepreg laminate, laid by AFP, is very small or negligible. Higher impacts on mechanical properties were also observed, which are displayed in Figure 2. According to Hsiao et al. [9], the influence of fibre waviness on the strength and stiffness of fibre composites can be very high. Waviness is defined as the ratio of amplitude to wavelength [10]. At a value of 0.15, the influence on the compression stiffness can lead to a reduction of 80% [9]. The evaluation criteria of wrinkles, fuzz balls, fibre fluff, binder residue, loose fibre, fibre folding, and twisted tow were not observed or were avoided in this work. Thus, they are not described in detail here.

### 1.3. Influencing of the Permeability of a DFP Preform

Permeability plays an important role for complete wetting of the filaments with a fluid resin by an infiltration process. It is defined here from the equations of fluid flow through porous media. For composite materials, it represents a measure of how easily they are impregnated by a fluid resin. There are several possibilities of how the permeability of DFP preforms is influenced in all three spatial directions, such as:

- Laying of gaps.
- Adaption of the ply structure/fibre orientation.
- Needling/tufting.
- Application of binder/binder yarns on the ply structure.
- Wettability of the fibre surface.

Since the influence of gaps on the permeability is already described in the literature as significant, we decided to consider this process parameter and link it to the influence on the mechanical properties. Aziz et al. [11] describe that the influence of gaps on permeability can be up to a factor of 5 regarding the correlation to a ply configuration without gaps. Since unidirectional laminates barely appear in practice, and a systematic analysis of the parameter could not be found in literature, the influence of 90° ply in the laminate was also chosen as an investigation parameter. The fact that no or little influence on the mechanical properties has been observed in prepreg materials [8] lead to the decision to analyse the influence of gaps in more detail. The influence of different binder systems was not considered in this work and was kept constant for all test series. The tufting of preforms can also achieve significant increases in permeability, which is associated with a reduction in flexural strength of up to 29% and 23% in the flexural modulus [12]. The effort of tufting is much higher than laying gaps and the decrease in mechanical properties is significant. Finally, the influence of gaps and fibre orientation on the permeability was investigated with respect to the influence on the mechanical properties of strength/stiffness in tension and bending.

The wettability of the fibre itself also plays an important role, but it was not a subject of the investigation in this study. The work focused on the method of determining infiltration times by a general approach over a whole preform. In [13], it is described that the permeability strongly depends on the fibre fluid combination and must be kept constant for comparative testing. Furthermore, it is demonstrated that the contact angle depends only on the fibre and not on the configuration of the preform. According to Hammond et al. [14], viscosity variations have little influence on the permeability measurements.

### 1.4. Scope

Compared to the state of the art, which provides complex and expensive robot-based or large gantry-based laying machines, the whole process of spreading and the layup of fixed tows was simplified. This was done to make the technology more applicable for a wider range of parts and decrease the manufacturing costs, especially for small- and medium-sized enterprises. The scope of the work was to analyse the infusion behaviour of this specific process and set the parameters in relation to the achievable mechanical values. As known from the state of the art, gaps and variation of the fibre angles within a preform can influence the permeability by several magnitudes and have small influences on the mechanical properties. They are also the parameters that can be simply varied because the machine technology directly provides the possibility to change them. All other known methods, e.g., stitching or additional binders, require extra process steps and additional machines. Therefore, the parameters of gaps and fibre orientation were chosen for this study. Furthermore, a simple method to predict the VAP infusion times of DFP preforms with various thicknesses and layer configurations was addressed. The method should provide the possibility to derive the infiltration time curves for specific material combinations by performing only a small number of infusions trials. The approach includes Darcy′s law for porous materials to develop a method for the prediction of VAP infusion times.

## 2. Materials and Methods

### 2.1. Materials Used for Sample Plates

Table 1 lists all the materials used in the study. All sample plates were manufactured using these three materials. The Hexion Epikote Resin TRAC 06720 binder system was applied based on the results from Helber et al. [1], where a good tow stabilization and a low deviation in the width of the fixed tow were observed. The binder showed a good impregnation behaviour of the fibres. Helber et al. [1] observed that the SGL SIGRAFIL^®^C T24-5.0/270-E100 showed good spreading behaviour too. The basic properties of the used materials are provided in Appendix A, Table A1, Table A2 and Table A3.

### 2.2. Manufacturing of Fixed Tow

**Figure 3:**
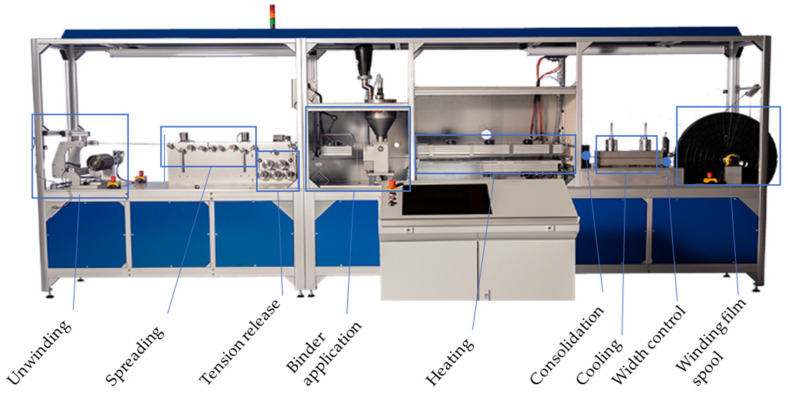
Spreading machine from M&A Dieterle GmbH to produce the fixed tow material.

To produce the fixed tows, the tows were spread to a width of 20 mm (±0.5) with a mechanical spreader and fixed by the binder. The powdered binder was applied to the spread tow and thermally activated using infrared lamps, consolidated, cooled, and ultimately wound onto a film spool. The used amount of binder was 8% for all specimens. For all fixed tows that were produced, the binder was activated at 110 °C. The properties of the fixed tow are shown in Table 2. In Figure 3, the used spreading machine is displayed. From left to right, it consists of an unwinding unit, followed by a spreading element and an arrangement of rollers to reduce the tension. The middle part starts with the binder application unit, where the powder binder is applied. After the binder application, the binder is thermally activated, and the fixed tow is consolidated. The last section cools down the fixed tow and controls the width using an edge detection sensor before winding it on the film spool.

### 2.3. Production of Preforms by DFP

**Figure 4:**
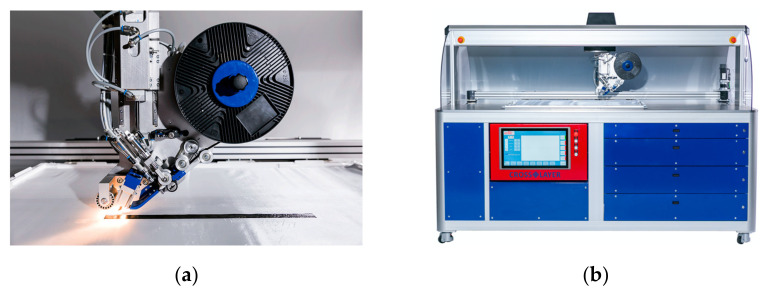
(**a**) Crosslayer placement head; (**b**) DFP Crosslayer machine from M&A Dieterle GmbH for the production of the preforms.

Figure 4 shows the placement head and the DFP machine, which has been used for the manufacturing of the preforms. The fixed tows were placed at a laying speed of 8.5 m/min and an IR halogen lamp output of 360 W. A maximum of 7 plies were placed on top of each other. To produce preforms with more than 7 plies, the ply configuration was divided into several sub-preforms and stacked manually afterwards. The preform size was 350 mm × 340 mm and the plies were placed on a plain weave glass fibre fabric, which served as the placement substrate. The substrate only served as a carrier for the placement process and was subsequently removed before the infusion. Plies without gaps were laid according to the fixed tow width of a 20 mm path spacing. Regarding plies with 2 mm gaps, a path spacing of 22 mm was used. Due to the programming of the paths of the fixed tows and the width of the fixed tows, the occurrence of overlaps was excluded. Wrinkles and tow twists were not observed.

### 2.4. Liquid Resin Infusion by the Vacuum-Assisted Process (VAP)

**Figure 5:**
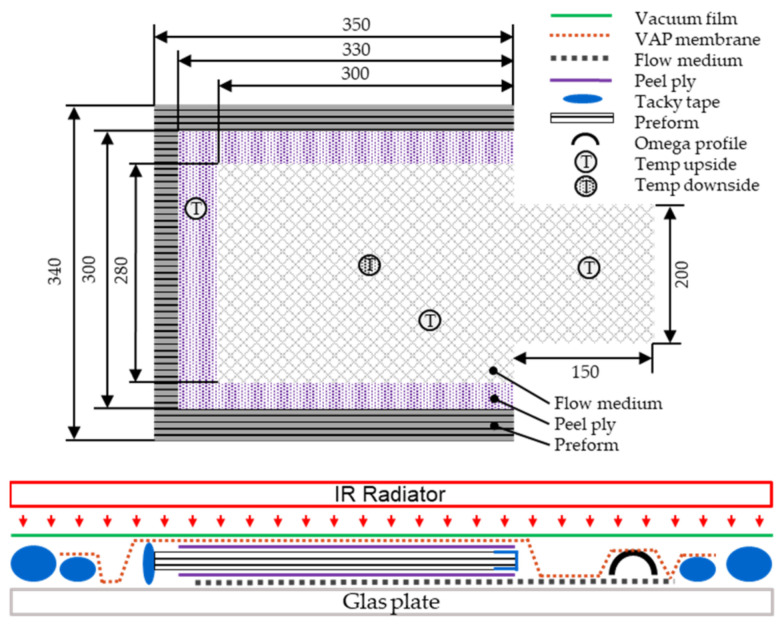
Schematic of the VAP infusion setup, preform size, blanks: flow medium and peel ply.

The VAP infusion is a patented variation of resin infusion, where a semipermeable membrane separates the vacuum outlet from the surface of the part. This creates a full vacuum gradient and continued degassing across the part surface, as opposed to only at the end-edge of the part in the traditional resin infusion [15]. For the investigation of the infiltration behaviour, the distance between the fixed tows (2 mm gaps and no gaps) and the number of plies and ply orientations of the laminate were varied. For this purpose, a test setup was chosen, in which the preforms were infiltrated on a glass plate. The preforms were infiltrated in the z-direction (out-of-plane) against gravity. The process parameters, listed in Table 3, were kept constant for the production of all test plates and were permanently measured and documented during the test. The temperature was monitored by means of type K temperature sensors at four measuring points, three on the top and one on the underside of the infiltration setup. A schematic of the setup is displayed in Figure 5. The temperature of the infusion setup was controlled by a regulated IR radiator (Trisk, type: 420,469, 3 × 1.5 kW) from the top. The temperature was measured by a pyrometer on the surface of the infusion setup. The parameters for infusion were set according to the technical data sheet of the manufacturer. The temperature setting of 35 °C provided a viscosity of 180 mPas with a reasonable pot life of 120 min for the infiltration process. In addition, the controlled infusion temperature ensured constant infusion parameters independent of the ambient temperature. The evacuated infusion setup was pre-heated 30 min before starting the infiltration.

**Figure 6:**
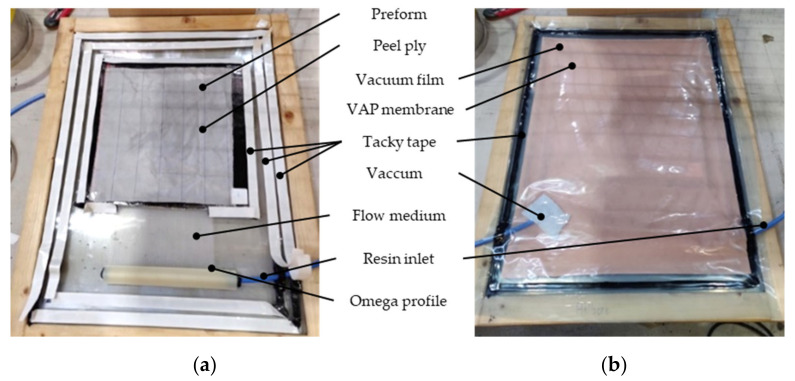
VAP Infusion setup: (**a**) preform and support material and sealings; (**b**) closed setup with VAP membrane, vacuum film, resin inlet, and vacuum tube, no vacuum applied yet.

The preforms were sealed at the resin inlet with an adhesive tape (Solvay, Flash Tape 2) and all around with an additional strip of Tacky Tape (Cytec, LTS90) sealing, to prevent the matrix system from flowing sideways to the upper side, and thus, creating dry spots inside the laminates. Figure 6a shows the inside of the infusion setup without the VAP membrane and without the vacuum film. In Figure 6b the closed setup is displayed with no vacuum applied yet.

### 2.5. Relation between Laminate Thickness and Infusion Time B-Factor

The Darcy approach was chosen to predict the infiltration time in the z-direction of a specific ply structure, see Equation (1a). It was chosen because in the field of composites, it is a very simple and common way to describe the fluid dynamics of a porous material or fabric, which should be wetted by a low viscous matrix. The approach gives an averaged behaviour over the measured area. It is also very useful for a comparison of different test methods or benchmark studies [16]. The Navier–Stokes and the Brinkman equations, suitable for open flow regions (e.g., in between yarns) and for porous regions (within a yarn or fibre), respectively, can be used. They can be combined to describe the flow of a fluid in a composite textile. Using the approach of these equations, microscopic and mesoscopic models of the preforms are necessary [13]. This would interfere with the scope of the work to provide a simple method for the prediction of infusion times.

Since the laminate thickness is very small, the influence of gravity on the pressure difference was neglected.

Darcy’s law [17]:

(1a) $$K_{z}=-\frac{q*\eta *\Delta z}{A*\Delta p}$$

(1b) $$q=\frac{A*z}{t}$$

(1c) $$t\left(z\right)=\frac{\eta }{K_{z}*\Delta p}*z^{2}$$

$$\mathrm{with}:\;\overset{\eta ,\;\Delta p,K_{z}=\mathrm{const}}{\to }$$

(2) $$\mathrm{follows}:\;t\left(z\right)=B*z^{2}$$

Definitions:

$K_{z}\;\left[m^{2}\right]$: Permeability in the z-direction (out of plane).

$q\;\left[\frac{m^{3}}{s}\right]$: Volume flow.

$z\;\left[m\right]$: Distance in the z-direction.

$t\;\left[s\right]$: Time.

$\eta \;\left[\frac{\mathrm{Ns}}{m^{2}}\right]$: Dynamic viscosity.

$\Delta p\;\left[\frac{N}{m^{2}}\right]$: Pressure difference.

$B\;\left[\frac{s}{m^{2}}\right]$: Material factor (with $\eta ,\;\Delta p,\;K_{z}\;=\;\mathrm{const}$.).

Equation (2) shows that the infiltration time t represents a parabola passing through the zero point. The function depends on the preform thickness *z* (*z*-direction) at constant viscosity η, differential pressure Δp, and permeability $K_{z}$. It follows that the infiltration time of a certain ply configuration increases quadratically with the thickness. By determining at least one base point, a curve can be generated from which the infiltration times can be derived for all thicknesses of the configuration. Infiltration times were measured to determine the curves. A material factor of *B* can be defined and calculated for the curves according to Equation (2). To determine the factor *B* and to validate the approach for a certain configuration, a minimum of 3 points were measured. The average value of *B* was calculated and used to define the curve of the corresponding configuration.

### 2.6. Measurement of Infiltration Time in the z-Direction

**Figure 7:**
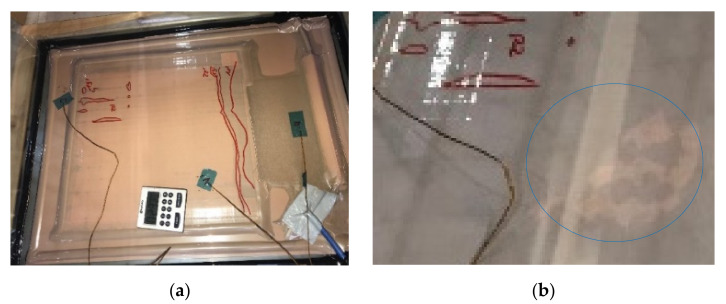
(**a**) VAP-Infusion–measurement of the infiltration time, wet spots, and flow front are marked, position of temperature sensors. (**b**) Infusion briefly before entire impregnation; the last dry spot is marked.

The infiltration time measurement started with the opening of the resin inlet and lasted until the complete impregnation of the preform. For this, the upper side of the VAP structure was observed to be fully impregnated. Figure 7a shows how the flow front spreads in the x- and y-directions. At the top left, it can be seen that the preform has already impregnated in the z-direction (circumferential markings on the left). The positions of the three temperature sensors on the upper side can be seen. The fourth sensor is centrally located under the infiltration plate. In Figure 7b, a last dry spot of the infusion process can be observed, briefly before complete impregnation. If an infiltration was not completed until the end of the pot life of the resin (still dry spots observable), the thickness of the corresponding ply configuration was stated as not infiltratable.

### 2.7. Ply Configuration of Sample Plates

**Figure 8:**
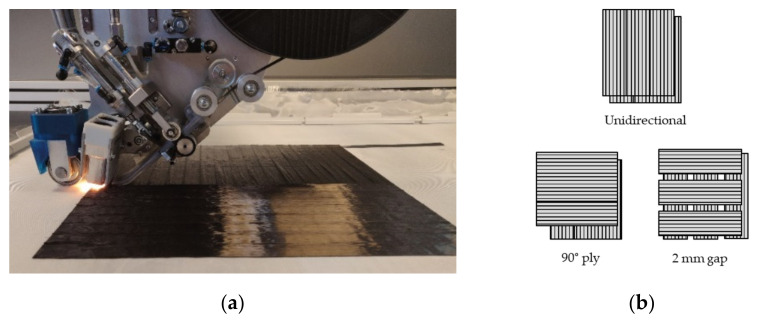
(**a**) DFP deposition of a preform configuration without gaps and 0° and 90° plies; (**b**) schematic of preform configurations.

**Figure 9:**
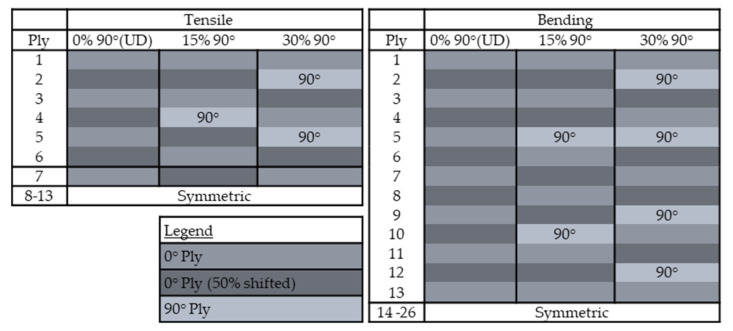
Ply configurations for the specimens for tensile and bending tests.

For the production of test specimens, laminates were produced that met the requirements of the respective norm with the corresponding thickness and ply structure according to Table 4. The deposition of the fixed tows and the schematic of the preform configuration is displayed in Figure 8. In order to determine the influence of the gaps and the ratio of 90° plies on the tensile and bending properties, the ply structures according to Figure 9 were chosen for the panel production. Identical ply structures were used for the two variants without a gap and with a 2 mm gap, in order to be able to compare the results. For adjacent unidirectional plies, the layup path was parallelly shifted by 50% of the path distance. The influence of 2 mm gaps and the percentage of 90° plies on the mechanical tensile and bending properties of the laminates using the DFP process was examined in detail.

### 2.8. Testing Methods

**Figure 10:**
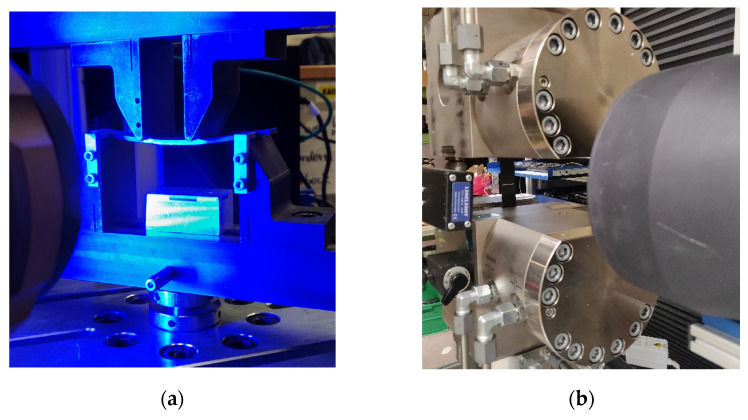
Test setups with video extensometer; (**a**) 4-point bending; (**b**) Tension.

All material characterisation tests were conducted according to the norms listed in Table 4. The used specimen geometries are displayed there as well. All conducted test series were completed after 5 valid measurements. A Hegewald and Peschke HP250 and HP25 testing machine was used for the tensile and bending tests, respectively. In Figure 10a the test setup for the 4-point bending test is displayed. Figure 10b shows the test setup for the tensile test.

### 2.9. Determination of the Fibre Volume Fraction

To determine the fibre volume fraction (FVF), 3 samples were taken from each plate. The samples were analysed according to the Norm EN 2564. These values were used to normalise all measured mechanical values to 55% FVF.

## 3. Results

### 3.1. Mechanical Values of Unidirectional Reference Laminate “No Gaps” and “No 90° Plies”

The measured characteristic values for the mechanical properties of strength and modulus for tension and bending are presented in Table 5. The values are separated into the two configurations of no gap and a 2 mm gap.

### 3.2. Mechanical Bending and Tensile Characteristics: Influence of the 2 mm Gaps and the Number of 90° Plies

The measured characteristic values for the mechanical values of bending strength, tensile strength, and stiffness are presented in Table 6. The values are separated into the two configurations of no gap and a 2 mm gap.

### 3.3. Infiltration Time

**Figure 11:**
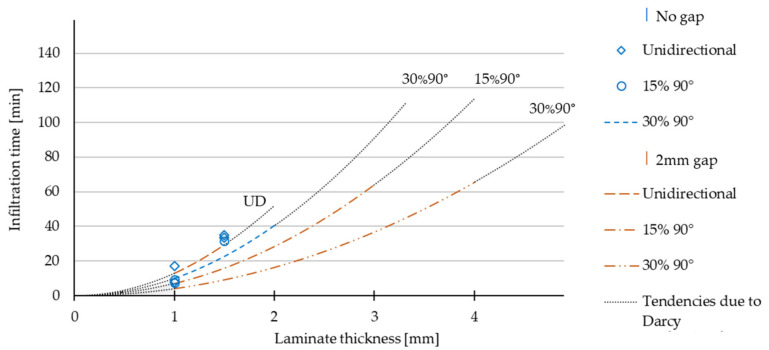
Infiltration times due to ply configuration, curves calculated by formula (2), based on the average B-factors of the different configurations in Table 7.

In Table 7, all measured infiltration times are displayed. For each ply configuration, a minimum of 3 values were measured. The factor B was calculated according to formula (2). A total number of 26 infusion trials were conducted. Fields with no value were spared out to reduce the number of tests or because the configuration was not infiltratable anymore. Table 7 shows 21 measured and calculated results where the infiltration time did not exceed the pot life of the resin (120 min). 5 values exceeded the pot life and are designated as not infiltratable. To determine the material factor B of a curve, the mean value of the B values of a configuration was used. Figure 11 shows the results of the calculated curves of the infiltration time depending on the ply configuration and the laminate thickness. The curves are displayed for the configuration of no gap with 30% 90° plies and the configurations with a 2 mm gap. The two configurations with no gap unidirectional and 15% 90° plies show a too high standard deviation for the calculated B-factor. Only the measured values are shown in the diagram. The curves are displayed up to the value of the maximum thickness, which was infiltrated. The dotted lines show the tendencies of the curves due to Darcy’s approach.

### 3.4. Laminate Quality and Cross-Sections

**Figure 12:**
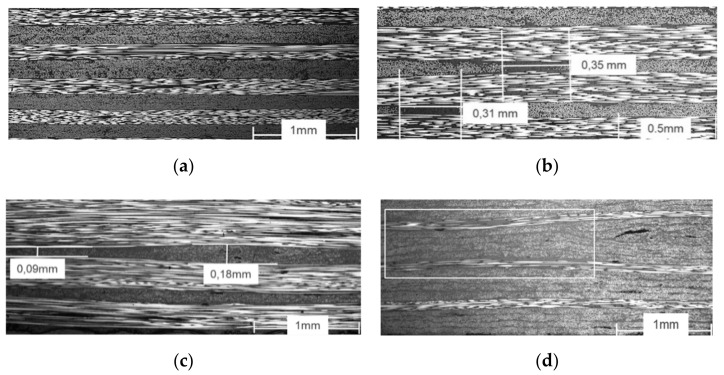
(**a**) Ply configuration [2 × 0°/2 × 90°], no gaps; (**b**) ply configuration 30% 90°, no gaps, resin accumulation due to spreading failure; (**c**) ply structure 15% 90°, no gaps, thickness variation due to inhomogeneous spreading; (**d**) ply structure 15% 90°, 2 mm gaps, gap => local undulation in 90° plies.

Representative micrograph analyses of the produced laminates are shown below, which allow an assessment of the laminate quality, such as the void content and fibre undulation. As can be seen in Figure 12a, homogeneous laminates with a low void content and fibre undulation were manufactured. It was found that the quality of the laminate is significantly related to the quality of the spreading. The figure also shows that within two layers of the same fibre orientation, no resin accumulation can be found, and an optical differentiation is barely visible. Figure 12b,c show possible defects that indicate spreading failures. Locally, resin accumulation (b) or thickness variations (c) can be observed. The measured width of the defects is between 0.31 and 0.35 mm and shows small introductions of undulation. The thickness variations range from 0.09 to 0.18 mm. The theoretical ply thickness after the spreading with the used parameters is 0.14 mm. Figure 12d shows a 2 mm gap and the resulting undulation of adjacent plies. The gap is marked by the square. It can be seen that the gap has a significant optical influence on the homogeneity of the ply structure. The figure also shows that the gap does not introduce any resin accumulation. The adjacent 90° plies fill in the gap completely and within the gaps undulate. On the right-hand side, a void can be observed, visible as a longish black spot.

The determined fibre volume fractions are displayed in Table 8. Each value stands for one plate, which was used to derive the specimens for the mechanical testing. The values are between 50.0% and 53.3%. The average value is 51.7% (±1.15). The standard deviations within the single test rows are between 0.4% and 3.8% of the mean values and can be seen as reliable. The values reveal that the FVF is higher for the unidirectional configurations with 53.0–53.3%. A difference between the no gap and 2 mm gap configuration cannot be seen. The values for the configuration with 90° plies are in the range of 50.0–51.4%.

## 4. Discussion

The most important findings and correlations are stated below in bullet points. The values are compared in relation to the reference unidirectional laminate values (Table 5).

### 4.1. Bending Characteristics: Influence of the 2 mm Gaps and the Number of 90° Plies

- First, 2-mm-wide gaps had no influence on the flexural modulus.
- The flexural strength was reduced by 3% if 2-mm-wide gaps were introduced.
- The flexural strength was reduced by 8% when 90° plies were inserted (ratio: 30%).

The results show that the 2 mm gaps had no influence on the flexural modulus of the tested samples. The reduction of the flexural strength was low with 3% in relation to the absolute value of a configuration without gaps. It can be explained by the small induced undulation due to the gaps. A smaller reduction of the flexural strength was seen with an increase of the 90° plies ratio than would be expected. Due to the position of the 0° plies in the outer area of the laminate, they can take up the resulting tensile and compressive forces in the case of a bending load. The standard deviations are on average 3.75% of the absolute values of the tensile strength and 2.75% of the absolute values of the tensile modulus. These values are typical for this kind of testing method. Subsequently, the measured values can be regarded as reliable.

### 4.2. Tensile Characteristics: Influence of the 2 mm Gaps and the Number of 90° Plies

- The tensile modulus was reduced by 2.6% when 2-mm-wide gaps were introduced.
- The tensile strength was reduced by 7% when 2-mm-wide gaps were introduced.
- The reduction in tensile strength and modulus correlated to the same extent with the percentage of 90° plies

The results show that the influence of the gaps on the tensile modulus was small. The influence on the tensile strength with a reduction of 7% was significantly greater. Croft et al. [8] found that the decrease in the ultimate strength is at 2.1% for a DFP laminate with a gap, whereas there was only one single gap introduced into the test specimen. This value is in the same dimension as the values measured within this study. It can be stated that the defects induced by gaps like undulation lead to earlier failure in the specimens. The standard deviations are on average 5% of the absolute values of the flexural strength and 4% of the absolute values of the flexural modulus. These values are typical for this kind of testing method. Subsequently, the measured values can be regarded as reliable. An influence of the number of 90° plies was not observed. Both configurations, no gaps and 2 mm gaps, showed the same drop, which correlates with the number of 90° plies, which were inserted.

To determine the influence of the process on the theoretical potential of the fibre-matrix combination, a calculation of the E-Modulus and strength, due to the CLT (classical laminate theory) [18], was made:

(3) $$E_{1}=E_{f}V_{f}+E_{m}\left(1-V_{f}\right)$$

(4) σ=E∗ε

Definitions:

$E_{1}\;\left[\frac{N}{{\mathrm{mm}}^{2}}\right]$: Modulus in the tensile direction.

$E_{f\;}\left[\frac{N}{{\mathrm{mm}}^{2}}\right]$: Modulus of the fibre.

$E_{m}\;\left[\frac{N}{{\mathrm{mm}}^{2}}\right]$: Modulus of the matrix.

$\sigma_{1max}\;\left[\frac{N}{{\mathrm{mm}}^{2}}\right]$: Strength in the tensile direction.

$V_{f}\;\left[-\right]$: Fibre volume fraction (FVF).

$\varepsilon \;\left[-\right]$: Strain.

$\sigma \;\left[\frac{N}{{\mathrm{mm}}^{2}}\right]$: Stress.

$E\;\left[\frac{N}{{\mathrm{mm}}^{2}}\right]$: Modulus.

$$E_{1}=150\;\mathrm{GPa}$$

$$\sigma_{1max}=2787\;\mathrm{MPa}$$

In Table 9, the values for the configuration with unidirectional fibres are calculated and compared with the measured values. As can be seen, the process loss for the unidirectional configuration with no gaps is 25% for the strength and 22% for the modulus. For the 2 mm gap configuration, the loss for the modulus is also 22% and the loss of strength is higher with 30%.

### 4.3. Influence of the Ply Configuration and Laminate Thickness on the Infiltration Time

Figure 11 shows that the influence of the gaps on the infusion speed for unidirectional laminates is small in relation to the configurations with 90° plies. The infusion speed for configurations without 2 mm gaps, but with an increasing 90° ratio, is still 33% for the configuration with 30% 90° plies compared to the unidirectional laminate. On average, the infusion time of a configuration with 2-mm-wide gaps is only 25% of the time compared to the configuration without gaps. The calculated tendency curve for the configuration of no gap with 30% 90° plies show that plates of 3 mm could theoretically be infiltrated within this configuration. However, in practice they were not. For the configurations of no gaps with no 90° plies and no gaps with 15% 90° plies, a maximum thickness of 1.5 mm was feasible. The calculated B factor, based on the measured infusion times for a 1 mm and 1.5 mm thickness, show high deviations. For this reason, no tendency curves were calculated. These deviations can be explained by different conditions or effects for configurations with no gaps, a thickness of less than 1.5 mm, and an amount of less than 15% 90° plies. These effects cannot be specified. The calculated B factors are more consistent within the other configuration of no gap with 30% 90° plies and all configurations with a 2 mm gap. The standard deviation for these configurations is between 10% and 33% of the mean value. The results lead to the conclusion that there are effects for unidirectional configurations and small thicknesses of DFP preforms. These have to be investigated in more detail and with alternative approaches. For the thicknesses, which come close to the point where there is no infiltration possible any more, the used approach is also not accurate. The accuracy of the approach increases for the configurations of a 2 mm gap with 15% 90° and 30% 90. It can be stated that the accuracy increases with an increasing number of 90°plies and implemented gaps.

## 5. Conclusions

Important influences of the process parameters of 2 mm gaps and number of 90° plies of the DFP process on the mechanical properties of the laminates were determined. Both parameters were systematically linked to their quantitative influence on the mechanical bending and tensile properties of the laminate and compared to the unidirectional reference laminate. Furthermore, the influence on the permeability of the laid preforms was investigated by measuring the infusion time during sample manufacturing. An outcome of this work is a simple guideline using the described methods, which can be used to influence and predict infusion times as well as to simultaneously predict the change in the mechanical properties. The results show that implementing 2 mm gaps between the fixed tows offers on average a 4 times faster infusion compared to configurations with no gaps. Increasing the number of 90° plies by 15% reduces the infusion time by a factor of 2. Only a small influence of the 2 mm gaps on the bending strength (3% reduction) was observed, while the influence on the tensile strength (7% reduction) was slightly higher. The influence on the stiffness was 2.7% for tensile and negligible for bending. The process loss was determined to 22% and 25% compared to the theoretical maximum tensile strength and stiffness. To determine the infiltration time of ply configurations with no gaps, a thickness less than 1.5 mm, and a low percentage of less than 15% 90° plies, the calculated curves are not very precise. The infusion behaviour within these configurations needs further investigation. The approach using Darcy′s law for the prediction of the infusion times could not be verified for all thicknesses within one configuration.

## Figures and Tables

**Table 1:** Used materials.

Material	Description
Fibre	SGL SIGRAFIL^®^C T24-5.0/270-E100
Binder	Hexion Epikote Resin TRAC 06720
Matrix	Hexion RIMR135/RIMH137
Substrate	Hexcel, HexForce 02116 1260 TF970, 105 g/m^2^, plain weave

**Table 2:** Fixed tow properties.

Fixed Tow	Unit	Value
Width	[mm]	20
Binder, one sided	[mass-%]	8
Tex fibre	[tex]	2400
Aerial weight, including binder	[g/m^2^]	89

**Table 3:** Infiltration process parameters and curing condition.

Parameter	Unit	Value
Temperature infusion	[°C]	35
Vacuum pressure, absolute	[mbar]	50
Temperature curing	[°C]	50
Curing time	[h]	16

**Table 4:** Norm for testing, specimen geometry, and test parameters.

Type of Value	Norm	Geometry (l × b × t)	Testing Length	Testing Speed	Strain Measurement
		[mm]	[mm]	[mm/min]	
Tensile	DIN-EN ISO 527-5	250 × 15 × 1	150	2	VE
Bending (4-point)	DIN-EN ISO 14125B	100 × 15 × 2	81	2	VE

VE: Video extensometer.

**Table 5:** Mechanical values of no gaps, 8 mass-% binder content-0% 90° plies; FVF normalised to 55%.

Type of Value	Unit	Value (Std Dev.)	Norm
0° Tensile strength	[MPa]	2079 (±133)	DIN-EN ISO 527-5
0° Tensile modulus	[GPa]	117 (±4)	DIN-EN ISO 527-5
0° Bending strength	[MPa]	1088 (±39)	DIN-EN ISO 14125B
0° Bending modulus	[GPa]	96 (±3)	DIN-EN ISO 14125B

**Table 6:** Mechanical values of tensile and bending in the 0° direction, 8 mass-% binder content, FVF normalised to 55%, standard deviation in () brackets.

		No Gap			2 mm Gap		
Type of Value	Unit	UD	15% 90°	30% 90°	UD	15% 90°	30% 90°
0° Tensile strength	[MPa]	2079 (±133)	1937 (±79)	1589 (±26)	1944 (±121)	1773 (±92)	1514 (±47)
0° Tensile modulus	[GPa]	117 (±4)	105 (±3)	88 (±1)	117 (±5)	98 (±4)	86 (±2)
0° Bending strength	[MPa]	1088 (±39)	1010 (±84)	977 (±37)	1032 (±53)	972 (±33)	956 (±55)
0° Bending modulus	[GPa]	96 (±3)	91 (±8)	70 (±2)	98 (±6)	88 (±2)	70 (±2)

**Table 7:** Results of the measured infiltration times and calculated material factor B.

	No Gap						2 mm Gap					
Thickness	UD	B	15% 90°	B	30% 90°	B	UD	B	15% 90°	B	30% 90°	B
[mm]	[min]	$\left[\frac{min}{mm^{2}}\right]$	[min]	$\left[\frac{min}{mm^{2}}\right]$	[min]	$\left[\frac{min}{mm^{2}}\right]$	[min]	$\left[\frac{min}{mm^{2}}\right]$	[min]	$\left[\frac{min}{mm^{2}}\right]$	[min]	$\left[\frac{min}{mm^{2}}\right]$
1	17.0	17.0	9.5	9.5	11.0	11.0	15.5	15.5	5.0	5.0	3.5	3.5
1	8.0	8.0	7.5	7.5	---		7.5	7.5	---		---	
1.5	79.0	35.1	71.0	31.6	24.0	10.7	36.0	16.0	---		---	
1.5	75.0	33.3	N/I		---		---		---		---	
2	N/I				35.0	8.8	N/I		40.0	10.0	14.0	3.5
2					N/I				---		27.0	6.8
3									57.0	6.3	27.0	3.0
4									N/I		59.0	3.7
Mean value		23.4		16.2		10.1		13.0		7.1		4.1
Std. dev.		11.3		10.9		1.0		3.9		2.1		1.4

N/I: Not infiltratable.

**Table 8:** Fibre volume fractions for the infiltrated plates used to determine the mechanical properties, standard deviation in () brackets.

		No Gap			2 Mm Gap		
Fibre Volume Fraction	Unit	UD	15% 90°	30% 90°	UD	15% 90°	30% 90°
1 mm plates tensile	[vol.-%]	53.0 (±0.2)	51.4 (±0.4)	50.4 (±0.2)	53.3 (±1.3)	51.4 (±0.6)	50.0 (±0.6)
2 mm plates bending	[vol.-%]	53.2 (±0.2)	50.8 (±0.3)	51.4 (±0.9)	53.2 (±1.2)	50.6 (±1.9)	51.0 (±0.7)

**Table 9:** Comparison of the measured unidirectional tensile strength and modulus to the theoretical value from the classical laminate theory (CLT).

		No Gaps			2 mm Gap	
Unidirectional	Unit	Measured	Process Loss	CLT	Measured	Process Loss
0° Tensile strength	[MPa]	2079	25%	2787	1944	30%
0° Tensile modulus	[GPa]	117	22%	150	117	22%

## Data Availability

Material support VAP membrane type CS/E from Composyst GmbH.

## Appendix A

**Table A1:** Basic values of C-Fibre: SGL SIGRAFIL^®^C T24-5.0/270-E100.

Property	Unit	Value
Filament Count	[-]	24,000
Tex	[tex]	1600
Density	[g/cm^3^]	1.79
Tensile strength	[GPa]	5.0
Tensile modulus	[GPa]	270
Strain max	[%]	1.9
Sizing		Epoxy
Sizing content	[m-%]	1.0

**Table A2:** Basic values of resin: Hexion RIMR135/RIMH137.

Property	Unit	Value
Density	[g/cm^3^]	1.19
Bending strength	[MPa]	110
Bending Modulus	[GPa]	2.95
Tensile strength	[MPa]	67.5
Compression strength	[MPa]	85
Strain max	[%]	7.5
Pot Life (35 °C)	[min]	120
Viscosity (35 °C)	[mPas]	180

**Table A3:** Basic values of powder binder: Hexion Epikote Resin TRAC 06720.

Property	Unit	Value
Bulk density	[g/cm^3^]	0.40
Particle size > 90 µm	[%]	5–20
Softening point	[°C]	100 ± 15
B-time at 150 °C	[s]	50–100
Reversible forming (90–110 °C)	[min]	0.1–3.0
Crosslinking irreversible (120 °C)	[min]	6
